# Long non-coding RNA MAFG-AS1 promotes proliferation and metastasis of breast cancer by modulating STC2 pathway

**DOI:** 10.1038/s41420-022-01043-z

**Published:** 2022-05-05

**Authors:** Shihao Di, Rumeng Bai, Die Lu, Chunni Chen, Tianshi Ma, Zigui Zou, Zhihong Zhang

**Affiliations:** 1 Department of Pathology, The First Affiliated Hospital of Nanjing Medical University, 300 Guangzhou road, 210029 Nanjing, China; 2 Department of Pathology, The First Affiliated Hospital of Soochow University, 899 Pinghai Road, 215006 Suzhou, China; 3 Department of Pathology, The Second Affiliated Hospital of Xi’an Jiaotong University (Xibei Hospital), 157 West 5th Road, 710004 Xi’an, China; 4 Department of Pathology, Zhejiang Provincial People’s Hospital & People’s Hospital of Hangzhou Medical College, 158 Shangtang Road, 310014 Hangzhou, China

**Keywords:** Breast cancer, Long non-coding RNAs

## Abstract

Breast cancer is the most common cancer worldwide. A number of studies proposed that long non-coding RNA plays an essential role in the regulation of invasion and metastasis of various forms of malignancy, including lung cancer, gastric cancer, and bladder cancer. In this study, a long non-coding RNA(LncRNA) MAFG-AS1 was explored in detail to understand the significance in the etiology of breast cancer. The results indicated that expression of LncRNA MAFG-AS1 in the breast cancer tissues was significantly higher than the adjacent normal breast tissues and elevated expression level of LncRNA MAFG-AS1 was correlated to the larger tumor size, negative expression of ER, PR and lymph node metastasis. The potency of breast cancer proliferation, invasion, and metastasis was inhibited in the absence of LncRNA MAFG-AS1. Mechanically, LncRNA MAFG-AS1 was mainly located in the cytoplasm. The downstream target gene of LncRNA MAFG-AS1 was STC2 which might promote cell proliferation and metastasis in breast cancer and this study provides a new potential therapeutic target for breast cancer.

## Introduction

Breast cancer (BC) is the most common malignancy worldwide and accounts for the largest proportion of cancer-related morbidity among women [1]. With the advancement of comprehensive treatments such as chemotherapy, endocrine therapy, and targeted therapy, the breast cancer-related mortality rate has been significantly reduced but still remains as one of the leading causes of cancer‐related death among women, and ranks 6th in cancer-related mortality in female patients in China [2]. While it is possible to cure breast cancer at its early stage, negligence in the self-examinations and clinical examinations leading to the diagnosis of advanced breast cancer. Even with the advancement in comprehensive therapies, the risks of recurrence and metastasis are still high [3].

The vast majority of the human genome is made up of non-coding RNA (ncRNA), apart from about only 2% protein-coding genes, which were found by gene chip technology and whole transcriptome sequencing [4–6]. Multiple evidence demonstrated that Long non‐coding RNAs (lncRNAs) play important roles in the development and progression of breast cancer. For example, the poor prognosis of triple-negative breast cancer (TNBC) was related to the elevated expression of LncRNA AFAP1-AS1 [7], ARNILA [8], ZNF469-3 [9], DANCR [10] and reduced expression of LncRNA H19 [11], GAS5 [12]. LncRNA MALAT1 regulated the critical pathway in TNBC progression and high expression of MALAT1 can upregulate the expression levels of c-MET and SOX4 by competitive binding with targeted mRNAs such as miR-34a/c-5p and miR-449a/b, then promoting proliferation and metastasis of tumor cells [13–15].

The MAF BZIP Transcription Factor G Antisense RNA 1 (MAFG-AS1) is a LncRNA with a length of 1914bp. Studies had proved that overexpression of MAFG-AS1 can upregulate the expression of polypyrimidine tract-binding protein 1 (PTBP1) through promoting its stability mediated by bound HuR, thus promoting the progression of bladder urothelial carcinoma (BUC) via regulation of the HUR/PTBP1 axis [16]. MAFG-AS1 can also serve as a molecular sponge of miR-744-5p to upregulate its nearby gene MAF bZIP transcription factor G (MAFG) in LUAD cells providing a potent and promising therapeutic target for LUAD patients [17]. Although many researchers had investigated the function and mechanisms of LncRNA MAFG-AS1 in tumors, its role in breast cancer still remained unclear. Results from this study revealed that the expression of LncRNA MAFG-AS1 in breast cancer tissues was evidently upregulated compared to the adjacent normal breast tissues. Stanniocalcin 2 (STC2) was the direct target gene of which might mediated the pro-progression role of LncRNA MAFG-AS1. Taken together, our study suggested that the clinical manifestation of MAFG-AS1, which can be used as a novel biomarker for diagnosis and potential therapeutic target for breast cancer.

## Results

### LncRNA MAFG-AS1 is upregulated in breast cancer tissues and associated with poor prognosis

**Fig. 1:**
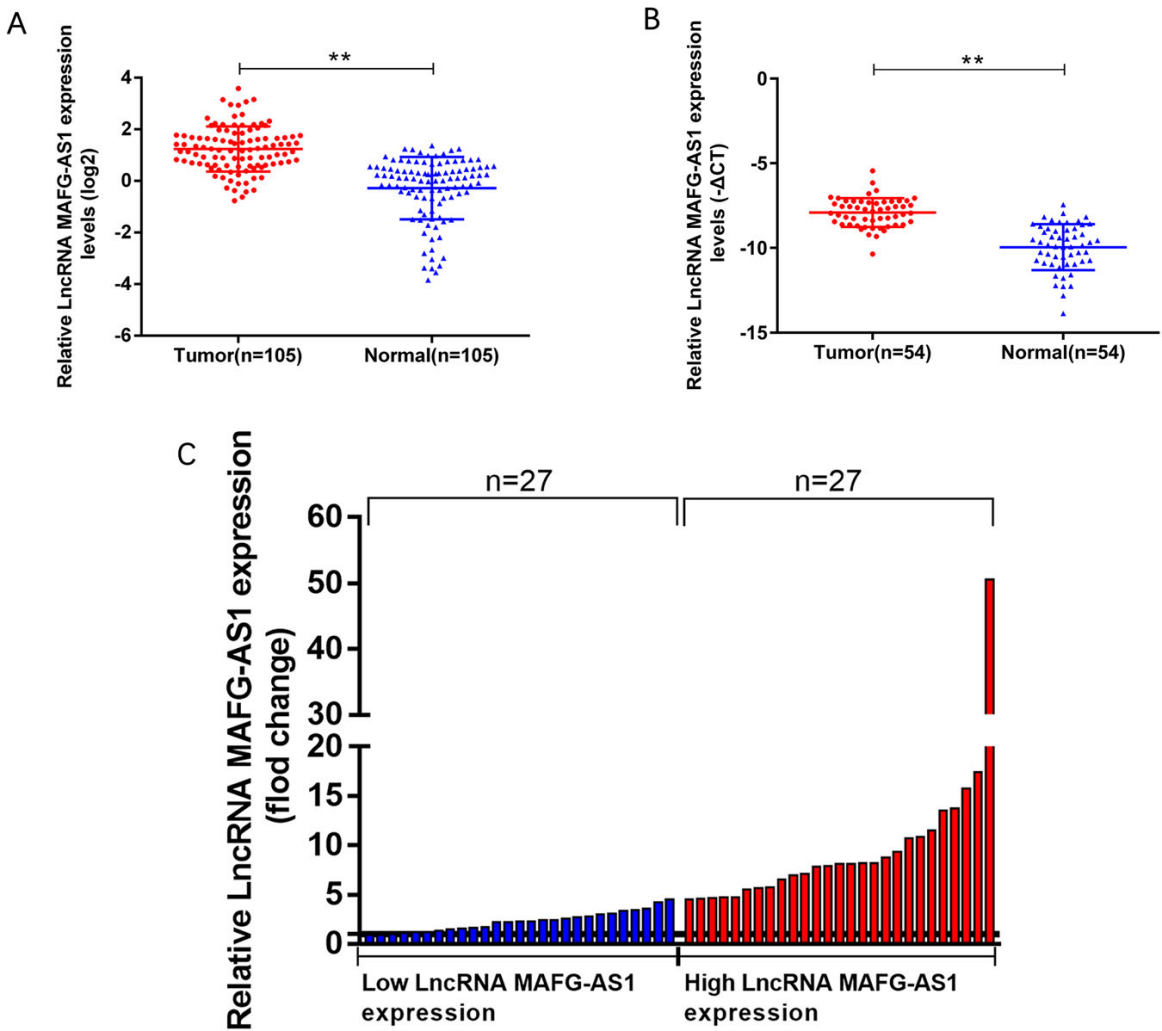
Relative expression of LncRNA MAFG-AS1 in BC tissues. **A** Data collected from TCGA database showed relative expression of LncRNA MAFG-AS1 in BC tissues (*n* = 105) and their corresponding nontumorous tissues (*n* = 105). **B**, **C** Relative LncRNA MAFG-AS1 expression in BC tissues (*n* = 54) compared with their adjacent normal breast tissues analyzed by qRT-PCR and tissues were divided into two groups according to the fold-change of LncRNA MAFG-AS1 expression. *P < 0.05, **P < 0.01.

In this study 105 patients with breast cancer were analyzed using bioinformatic tool “TANRIC” (http://ibl.mdanderson.org/tanric/_design/basic/main.html), the expression level of MAFG-AS1 in breast cancer tissues was significantly higher than the adjacent normal breast tissues (Fig. 1A). To further confirm this finding, we conducted qRT-PCR analysis in 54 pairs of breast cancer samples, and MAFG-AS1 expression level was significantly higher compared to the adjacent normal breast tissues (Fig. 1B). Then these 54 breast cancer samples were categorized into upregulated group (*n* = 27) and downregulated group (*n* = 27) based on qRT-PCR results (Fig. 1C) to explore the relationship between LncRNA MAFG-AS1 and clinicopathological characteristics. The results indicated that the upregulation of LncRNA MAFG-AS1 was related to the larger tumor size (*p* = 0.033), negative expression of ER (*p* = 0.040), PR (*p* = 0.024) and lymph node metastasis (*p* = 0.028). We could not find any effect of MAFG-AS1 with age (*p* = 0.780), Her2 (*p* = 0.248) and histological grade (*p* = 0.413) (Table 1).

**Table 1:** Correlation between LncRNA MAFG-AS1 expression and clinicopathological characteristics of BC patients (*n* = 54).

Characteristics	*N* (%)	LncRNA MAFG-AS1		*P* value
		Low expression group (*n* = 27)	High expression group (*n* = 27)	
**Age(years)**				
≤50	21 (38.9)	10	11	0.780
>50	33 (61.1%)	17	16	
**Tumor size**				
<2 cm	25 (46.3%)	8	17	0.033*
2–5 cm	27 (50.0%)	18	9	
>5 cm	2 (3.7%)	1	1	
**ER status**				
Negative	17 (31.5%)	12	5	0.040*
Positive	37 (68.5%)	15	22	
**PR status**				
Negative	20 (37.0%)	14	6	0.024*
Positive	34 (63.0%)	13	21	
**Her2 status**				
Negative	36 (66.7%)	16	20	0.248
Positive	18 (33.3%)	11	7	
**Lymph node metastasis**				
No	24 (44.4%)	8	16	0.028*
Yes	30 (55.6%)	19	11	
**Histological grade**				
I-II	29 (53.7%)	13	16	0.413
III	25 (46.3%)	14	11	

^*^P < 0.05 was considered significant.

All together our results indicated that LncRNA MAFG-AS1 was closely related to the prognosis of breast cancer patients, which can potentially be used as a biomarker for the breast cancer and metastasis.

### Functional measurement of LncRNA MAFG-AS1 in breast cancer cell lines

#### LncRNA MAFG-AS1 promotes proliferation of the breast cancer cells in vitro

**Fig. 2:**
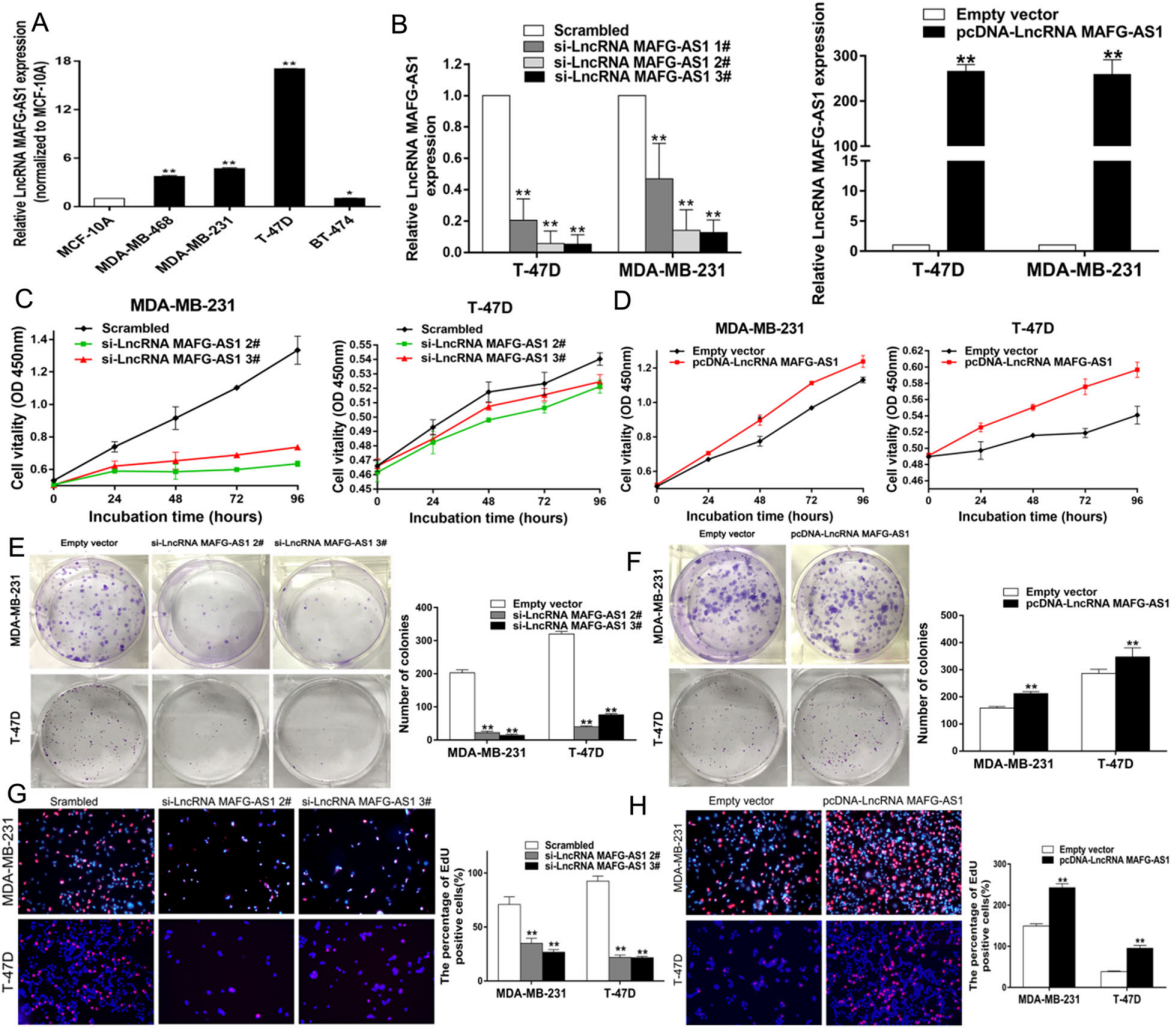
LncRNA MAFG-AS1 promotes BC cell-proliferation in vitro. **A q**RT-PCR assay examined LncRNA MAFG-AS1 expression in normal breast epithelial cell line (MCF-10A) and BC cell lines. **B** Left: qRT-PCR analysis of LncRNA MAFG-AS1 expression in BC cells transfected with control (scrambled), si-LncRNA MAFG-AS11#, 2#,3#. Right: Relative expression of LncRNA MAFG-AS1 in BC cells transfected with empty vector and pcDNA-LncRNA MAFG-AS1. **C**, **D** CCK-8 assays were performed to determine the viability of BC cells treated with si-LncRNA MAFG-AS1 or pcDNA-LncRNA MAFG-AS1. **E**, **F** Colony formation assays were used to detect the proliferation of si-LncRNA MAFG-AS1 or pcDNA-LncRNA MAFG-AS1-transfected BC cells and colonies were counted and captured. **G**, **H** Proliferous BC cells were displayed by EdU immunostaining assays. EdU positive cells were counted and captured. Values are shown as the mean ± s.d in three independent experiments. *P < 0.05, **P < 0.01.

In order to evaluate the biological function of LncRNA MAFG-AS1 in breast cancer cell lines, the expression levels of LncRNA MAFG-AS1 were examined in four different breast cancer cell lines (MDA-MB-468, MDA-MB-231, T-47D, BT-474) and healthy breast cell line (MCF-10A) by qRT-PCR. The quantitative results demonstrated that expression levels of LncRNA MAFG-AS1 elevated evidently in all four breast cancer cell lines compared to MCF-10A especially in MDA-MB-231 and T-47D which were selected as the experimental subjects in the following studies (Fig. 2A). First, three siRNAs targeted LncRNA MAFG-AS1 were designed and transfected into cell lines MDA-MB-231 and T-47D, and all three siRNAs were able to reduce the expression levels of LncRNA MAFG-AS1 in the experimental cell lines compared with the control group (Fig. 2B). Moreover, since the transfection efficiency of si-LncRNA lncRNA MAFG-AS1 #2, and #3 were relatively higher, they were chosen for the next confirmatory test. Next eukaryotic expression plasmids of LncRNA MAFG-AS1 were constructed and the following transfection assay demonstrated that the expression levels of LncRNA MAFG-AS1 were elevated distinctly in MDA-MB-231, and T-47D cell lines. CCK-8, colony formation assays and EdU assays were used to verify the regulating effect of LncRNA MAFG-AS1 for breast cancer cell proliferation. CCK-8 from these assays revealed that the absence of LncRNA MAFG-AS1 in MDA-MB-231 and T-47D could suppress the breast cancer proliferation activity dramatically (Fig. 2C), and over-expressing LncRNA MAFG-AS1 could promote the breast cancer cells proliferation activity clearly (Fig. 2D). As the results of colony formation assays demonstrated that knockdown of LncRNA MAFG-AS1 in MDA-MB-231 and T-47D could suppress the breast cancer colony viability visibly (Fig. 2E), while over-expressing LncRNA MAFG-AS1 could promote the breast cancer cells proliferation activity overtly (Fig. 2F). Similarly, results from EdU assays showed the inhibition of MDA-MB-231 and T-47D proliferation in the absence of MAFG-AS1 (Fig. 2G), while the proliferation rate was accelerated when MAFG-AS1 was overexpressed (Fig. 2H). Altogether, our results demonstrated that LncRNA MAFG-AS1 can modulate the proliferation of breast cancer cells.

#### Downregulation of LncRNA MAFG-AS1 triggers phase G1 stasis and apoptosis of breast cancer

**Fig. 3:**
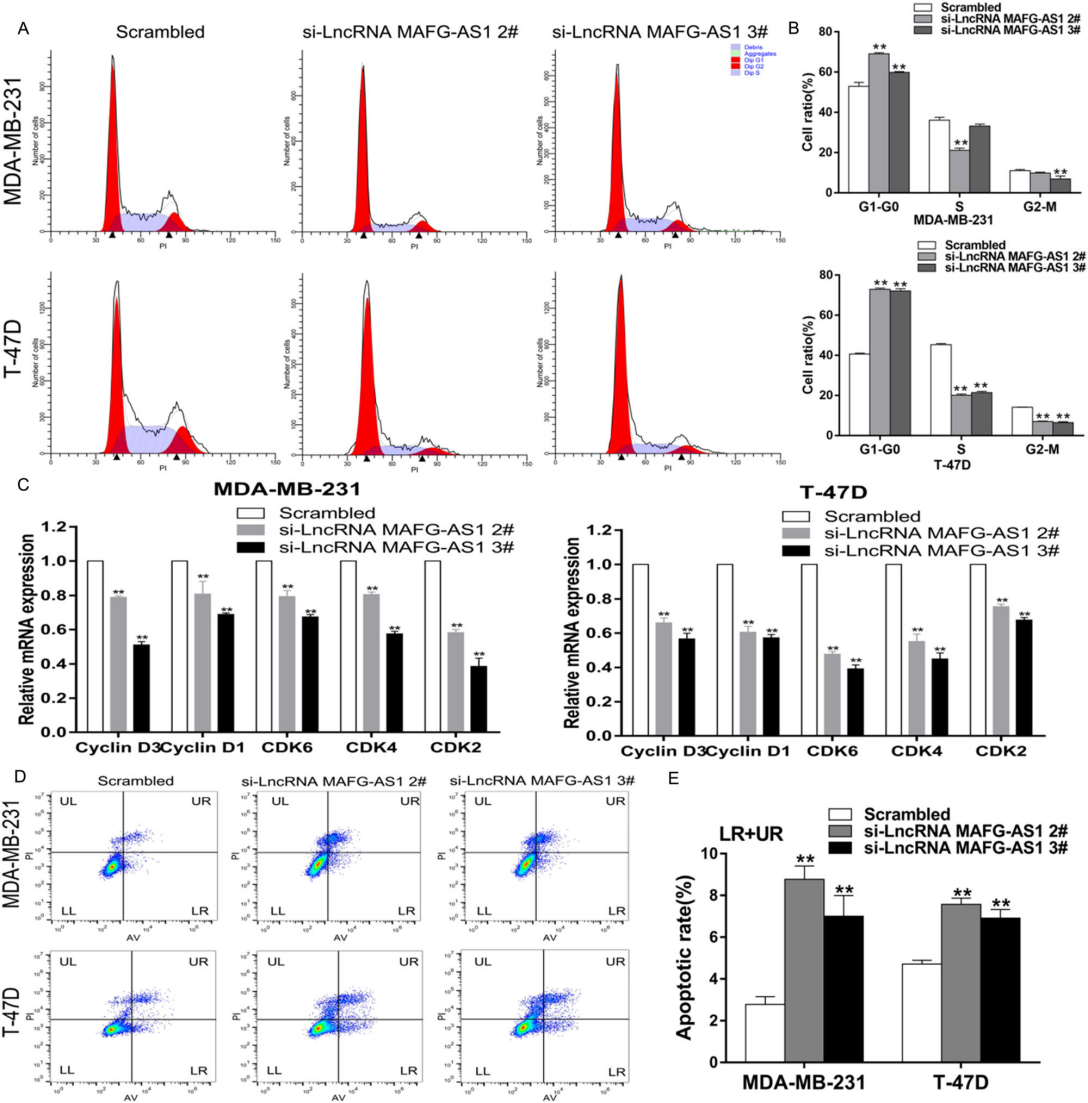
Inhibition of LncRNA MAFG-AS1 induces G1 arrest and apoptosis in BC cells. MDA-MB-231 and T-47D cells were transfected with si-LncRNA MAFG-AS1 or scrambled. **A**, **B** Cell cycle was determined in BC cells. **C** qRT-PCR analysis of CyclinD3, CyclinD1, CDK6, CDK4, CDK2 in BC cells. **D**, **E** Apoptotic rates of cells were tested by flow cytometry. Values are shown as the mean ± s.d in three independent experiments. *P < 0.05, **P < 0.01.

Cell cycle and apoptosis are the critical factors in the regulation of cell proliferation. We evaluated the cell cycle events and apoptosis by the flow-cytometry analysis. We observed that the cell cycle was ceased at phase G1-G0 in MDA-MB-231 and T-47D cells in the absence of LncRNA MAFG-AS1 (Fig. 3A, B). Moreover, qRT-PCR results demonstrated that expression levels of G1 phase-related genes (CyclinD3, CyclinD1, CDK6, CDK4, CDK2) declined after knocking down of LncRNA MAFG-AS1 in MDA-MB-231 and T-47D (Fig. 3C). Apoptosis assay with flow cytometry suggested that the ratio of apoptotic cells elevated evidently in the absence of LncRNA MAFG-AS1 in MDA-MB-231 and T-47D (Fig. 3D, E). Altogether, we conclude that the knockdown of LncRNA MAFG-AS1 suppressed breast cancer cell proliferation by triggering cell cycle arrest at G1 phase and augment of cell apoptosis.

#### Knockdown of LncRNA MAFG-AS1 suppresses breast cancer invasion and metastasis

**Fig. 4:**
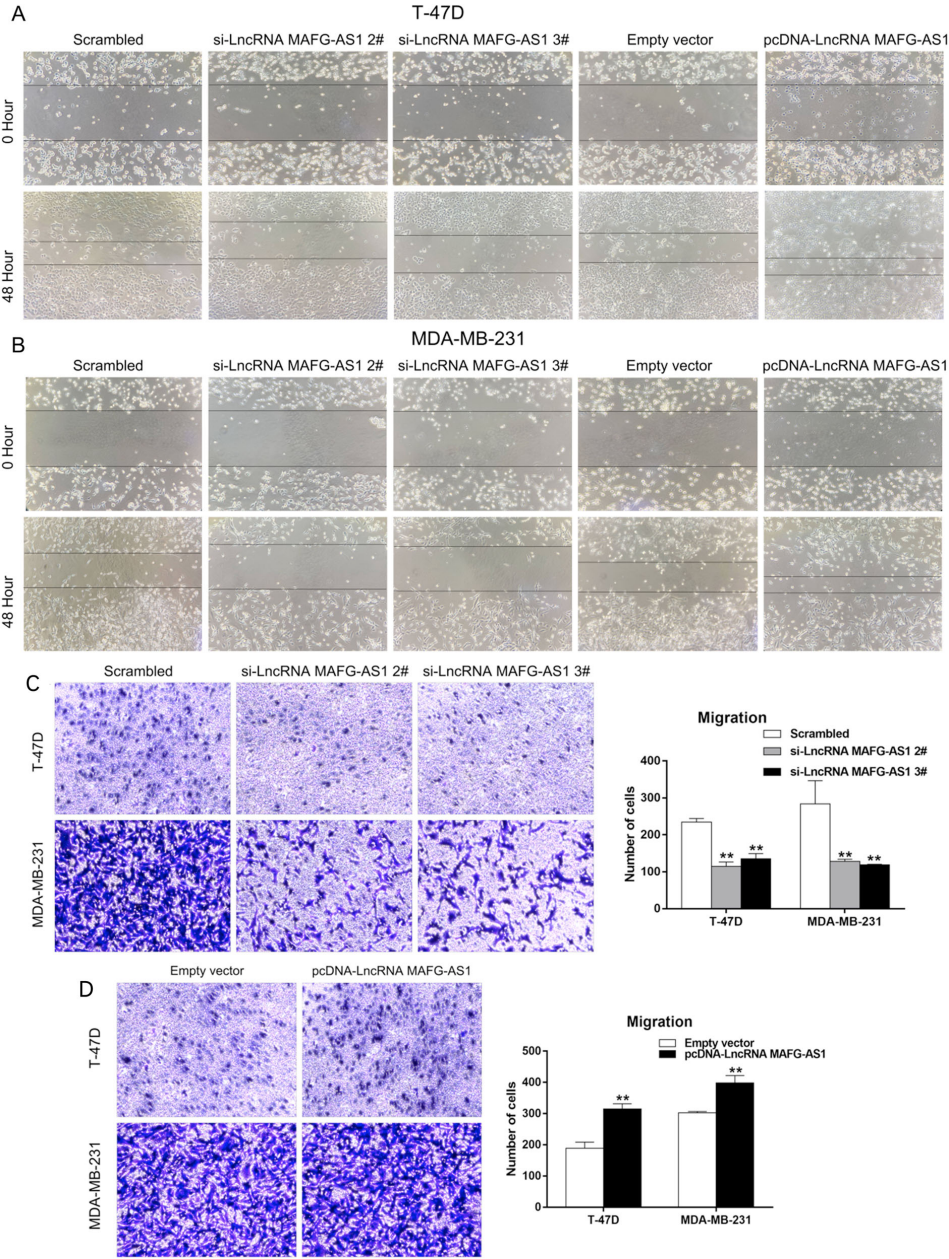
Effects of LncRNA MAFG-AS1 on BC migration in vitro in MDA-MB-231 and T-47D cells. **A**, **B** Wound-healing assays were used to investigate the migratory ability of BC cells. **C**, **D** Transwell migration assays were used to investigate the changes in migratory abilities of BC cells. *P < 0.05 and **P < 0.01.

Cancer cells migration and invasion are critical steps for tumor infiltration and metastasis. We observed that downregulation of lncRNA MAFG-AS1 could inhibit the migration ability of breast cancer cells and overexpression of MAFG-AS1 showed the opposite effect by the wound-healing assays (Fig. 4A, B). Next, we explored the migration and invasion abilities of breast cancer cell through Transwell migration and invasion assays. We found an increased number of cells transmitting the basement membrane compared to the control group, indicating that lncRNA MAFG-AS1 might promote the migration and invasion of breast cancer cells (Fig. 4C, D, Supplementary fig 1A, B), and hence suppression of the expression of MAFG-AS1 has important clinical application in the etiology of tumorigenesis. Our results suggested that knockdown of LncRNA MAFG-AS1 suppressed migration and invasion abilities of the breast cancer cell.

#### The LncRNA MAFG-AS1 promotes breast cancer proliferation in vivo

**Fig. 5:**
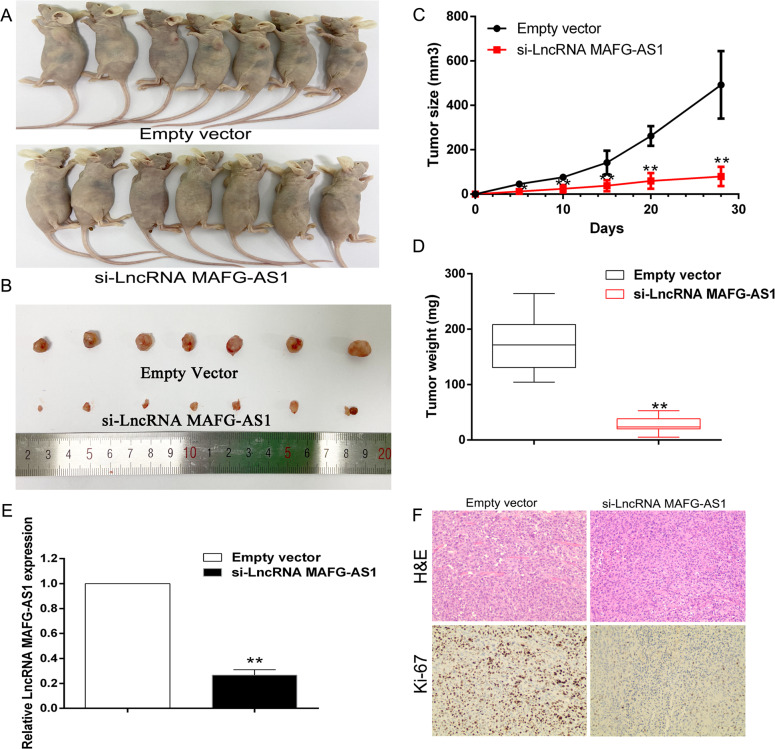
LncRNA MAFG-AS1 knockdown inhibits tumorigenesis of BC cells in vivo. **A**, **B** Empty vector or si-LncRNA MAFG-AS1 was transfected into T-47D cells, which were injected in the BALB/c-nude mice (*n* = 7), respectively. Tumors before and after carrying from the nude mice. **C** Tumor volumes were calculated after injection every 5 days. Points, mean (*n* = 7); bars indicated SD. **D** Tumor weights were represented as means of tumor weights ± SD. **E** qRT-PCR was used to detect the average expression of LncRNA MAFG-AS1 in xenograft tumors (*n* = 7). **F** The tumor sections were under H&E staining and IHC staining using antibodies against Ki-67. *P < 0.05, **P < 0.01.

In order to further evaluate the impact of LncRNA MAFG-AS1 on breast cancer cells proliferation, a xenograft model in nude mice was designed. We transfected T-47D cells with si-LncRNA MAG-AS1 and empty vector which were then inoculated into 4-week-old female nude mice either subcutaneously, or under the axilla separately. We recorded the size of tumor size immediately after the tumor formation. Finally, we euthanized the animals, and the tumor images were collected, then the size and weight of the tumor were measured (Fig. 5A). Tumor size and tumor weight indicated that knockdown of LncRNA MAFG-AS1 dramatically influenced in reducing the mean weigh of tumors compared to the control group (Fig. 5B–D). Furthermore, qRT-PCR results suggested that knockdown of LncRNA MAFG-AS1 suppressed the expression level of LncRNA MAFG-AS1 in tumors compared to the control group (Fig. 5E). Immunohistochemistry results indicated that Ki-67 expression in the absence of LncRNA MAFG-AS1 was much weaker compared to the control group (Fig. 5F).

### The mechanisms of LncRNA MAFG-AS1 modulating breast cancer cells proliferation and metastasis

#### The lncRNA MAFG-AS1 modulates its downstream target STC2

**Fig. 6:**
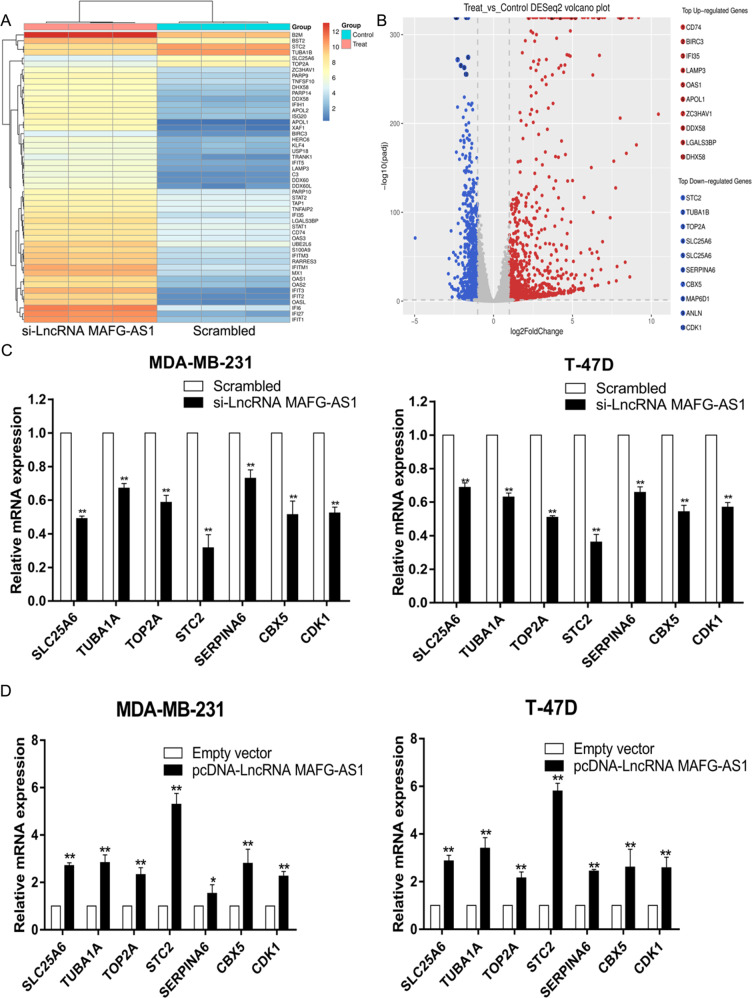
STC2 is a downstream target gene regulated by LncRNA MAFG-AS1. **A** Mean centered, hierarchical clustering of transcripts altered in scrambled siRNA treated cells and si-LncRNA MAFG-AS1 treated cells, with three repeats. **B** The top ten of upregulation or downregulation genes after si-LncRNA MAFG-AS1 in T-47D cells. **C**, **D** qRT-PCR analysis in si-LncRNA MAFG-AS1 treated or pcDNA-LncRNA MAFG-AS1 treated BC cells showed altered mRNA level of genes involved in cell apoptosis and migration upon LncRNA MAFG-AS1 depletion. Values are shown as the mean ± s.d in three independent experiments. *P < 0.05, **P < 0.01.

The altered gene expression profile after silencing the LncRNA MAFG-AS1 was evaluated using the transcriptome high-throughput sequencing to screen targeted gene regulated by LncRNA MAFG-AS1 (Fig. 6A). The screening result revealed the change in the expression pattern of 2237 genes (Fold Change>2, *P* value < 0.05) after silencing LncRNA MAFG-AS1 in T-47D cells. This included the 1387 upregulated genes, and 850 downregulated genes (Fig. 6B). Next qRT-PCR indicated that expression levels of STC2, TUBA1A, TOP2A, SLC25A6, SERPINA6, CBX5, CDK1 reduced apparently in T-47D and MDA-MB-231 cells after knockdown of LncRNA MAFG-AS1 which were conforming to the observations of high-throughput sequencing analysis, while over-expressing LncRNA MAFG-AS1 upregulated the expression of genes mentioned above (Fig. 6C, D). Also, we found that downregulation of STC2 was most prominent in T-47D and MDA-MB-231 cells after knocking down of LncRNA MAFG-AS1. Altogether, these results suggested that STC2 might be an important downstream gene regulated by LncRNA MAFG-AS1.

#### STC2 participates in the process of LncRNA MAFG-AS1 mediated proliferation

**Fig. 7:**
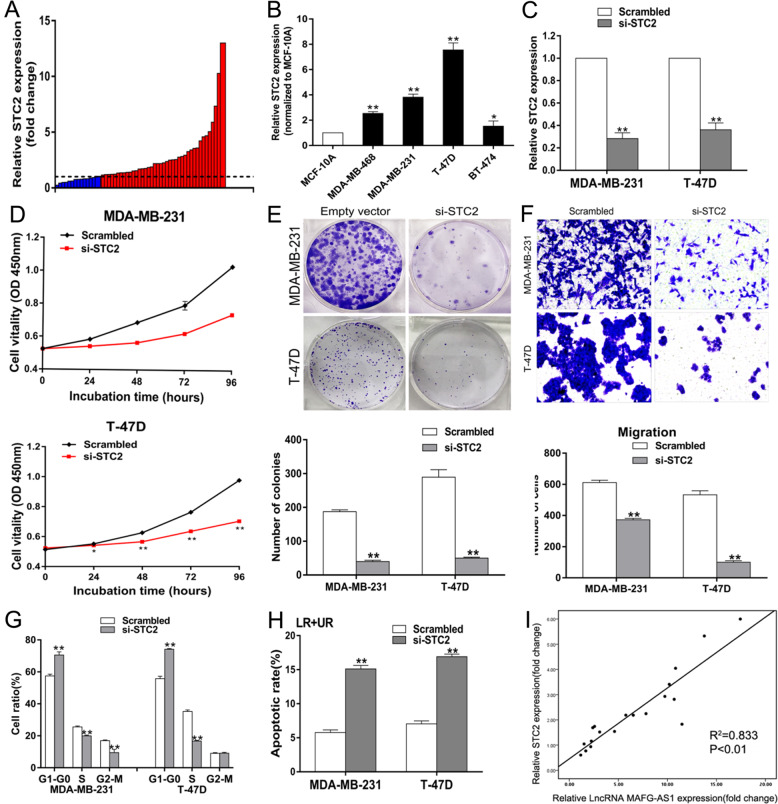
Downregulation of STC2 inhibits BC cells proliferation and is involved in the oncogene function of LncRNA MAFG-AS1. **A** Relative expression of STC2 in BC tissues (*n* = 54) and the paired noncancerous tissues (*n* = 54) analyzed by qRT-PCR and normalized to GAPDH. **B** qRT-PCR assay examined STC2 expression in normal breast epithelial cell line (MCF-10A) and BC cell lines. **C** qRT-PCR analysis of STC2 expression in BC cells transfected with control (scrambled) and si-STC2. **D** CCK8 assays were performed to determine viability of BC cells treated with the control and si-STC2. **E** Colony formation assays were used to detect the proliferation of si-STC2-transfected BC cells. Colonies were counted and captured. **F** Transwell assays were conducted to evaluate migration of BC cells. **G**, **H** Cell cycle and apoptosis of BC cells were investigated by flow cytometry. **I** Analysis of the relationship between STC2 and LncRNA MAFG-AS1 expression. *P < 0.05, **P < 0.01.

In order to study the specific function of STC2 in breast cancer, we studied the expression profiles of STC2 in 54 breast cancer tissues by using qRT-PCR. Results revealed that the expression level of STC2 in breast cancer tissues was distinctly higher compared to the precancerous lesions (Fig. 7A). We detected STC2 expression in four different breast cancer tissues and normal breast cell lines, whose results showed that the expression level of STC2 in 4 breast cancer tissues was overtly higher compared to MCF-10A (Fig. 7B). Then we designed and synthesized the siRNA of STC2 to verify the function of STC2 in breast cancer (Fig. 7C). We utilized CCK-8 and colony formation assays to dissect the knockdown of STC2 and found to be suppressed the breast cancer proliferation dramatically (Fig. 7D, E). It was found that knockdown of STC2 suppressed the migration of breast cancer cells by Transwell migration assays (Fig. 7F), and that knockdown of STC2 suppressed breast cancer cells proliferation by inducing cells into cell cycle arrest at G1 phase and augment of cell apoptosis (Fig. 7G, H). Our results confirmed that suppressing the expression of either STC2 or lncRNA MAFG-AS1 had the same impact on breast cancer proliferation and metastasis that were dramatically reduced.

**Fig. 8:**
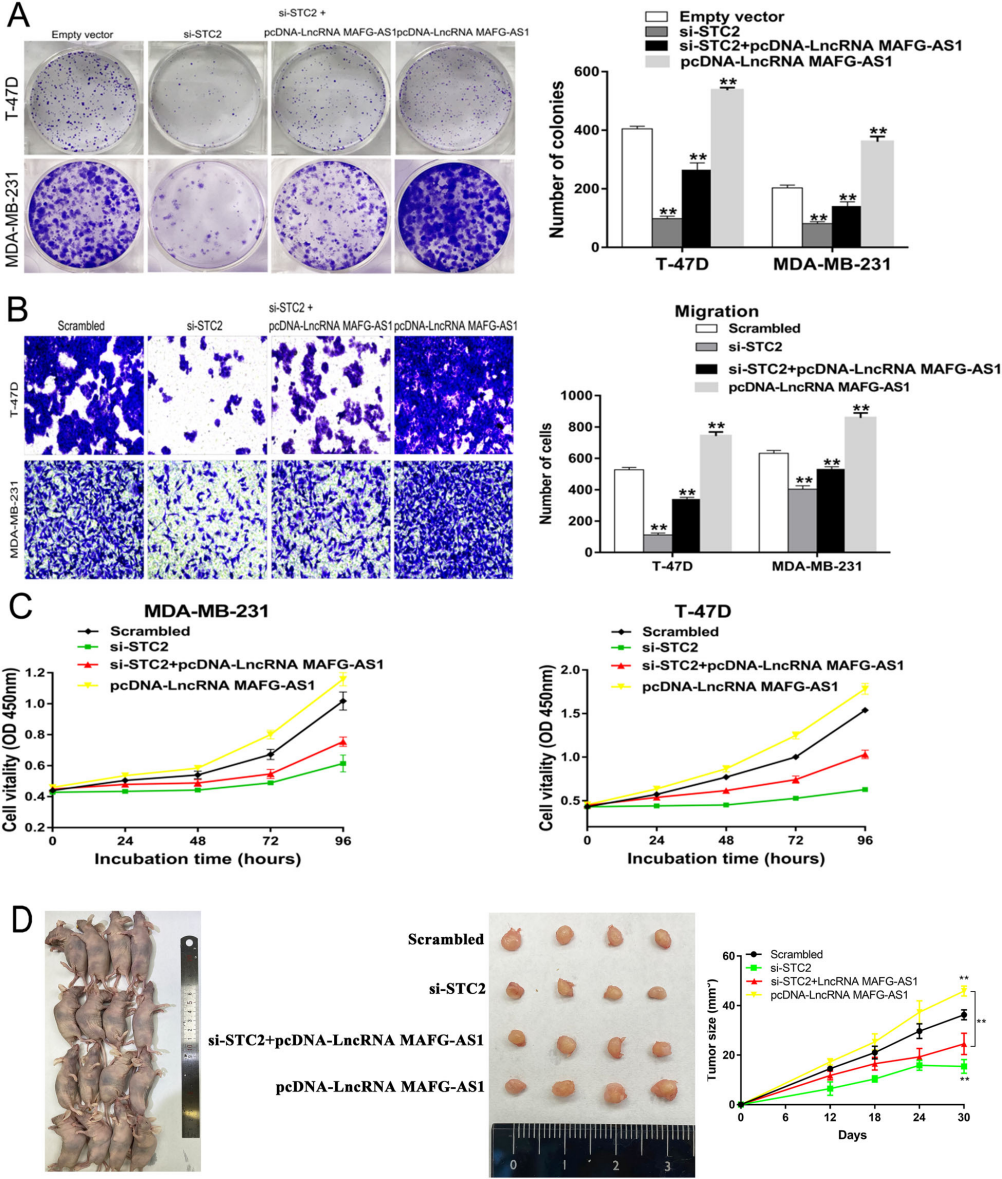
Downregulation of STC2 inhibits BC cells proliferation and is involved in the oncogene function of LncRNA MAFG-AS1. **A** Colony formation assays were used to determine the proliferation for si-STC2 and pcDNA-LncRNA MAFG-AS1 co-transfected BC cells. **B** Transwell assays were used to evaluate the migration for si-STC2 and pcDNA-LncRNA MAFG-AS1 co-transfected BC cells. **C** CCK-8 assays were used to determine the cell viability for si-STC2 and pcDNA-LncRNA MAFG-AS1 co-transfected T-47D cells. **D** Nude mouse subcutaneous tumorigenesis assays were used to determine the cell-proliferation ability for si-STC2 and pcDNA-LncRNA MAFG-AS1 co-transfected in BC cells, the right was tumor volume curve. Values are shown as the mean ± s.d in three independent experiments. *P < 0.05, **P < 0.01.

We further evaluated the connection between LncRNA MAFG-AS1, and STC2 using qRT-PCR for detecting the expression levels of LncRNA MAFG-AS1 and STC2 in breast cancer tissues and in adjacent normal breast tissues. We found a positive correlation between the expression levels of LncRNA MAFG-AS1 and STC2 (Fig. 7I). For testing whether STC2 participated in promoting the breast cancer cell proliferation and metastasis mediated by LncRNA MAFG-AS1, rescue experiments were carried out. pcDNA-LncRNA MAFG-AS1 and si-STC2 were co-transfected into MDA-MB-231 and T-47D cells. The results of CCK-8 and Transwell migration assays demonstrated that knockdown of STC2 in MDA-MB-231 and T-47D cells could partially rescue the increased cell proliferating migrating viabilities induced by over-expressing LncRNA MAFG-AS1 (Fig. 8A–C), and these results were consistent with experiments in vivo (Fig. 8D). Hence, all the results proved that LncRNA MAFG-AS1 could promote breast cancer proliferation and metastasis may partially by up-regulating the expression of STC2.

#### EMT process may play an important role in breast cancer invasion and migration

**Fig. 9:**
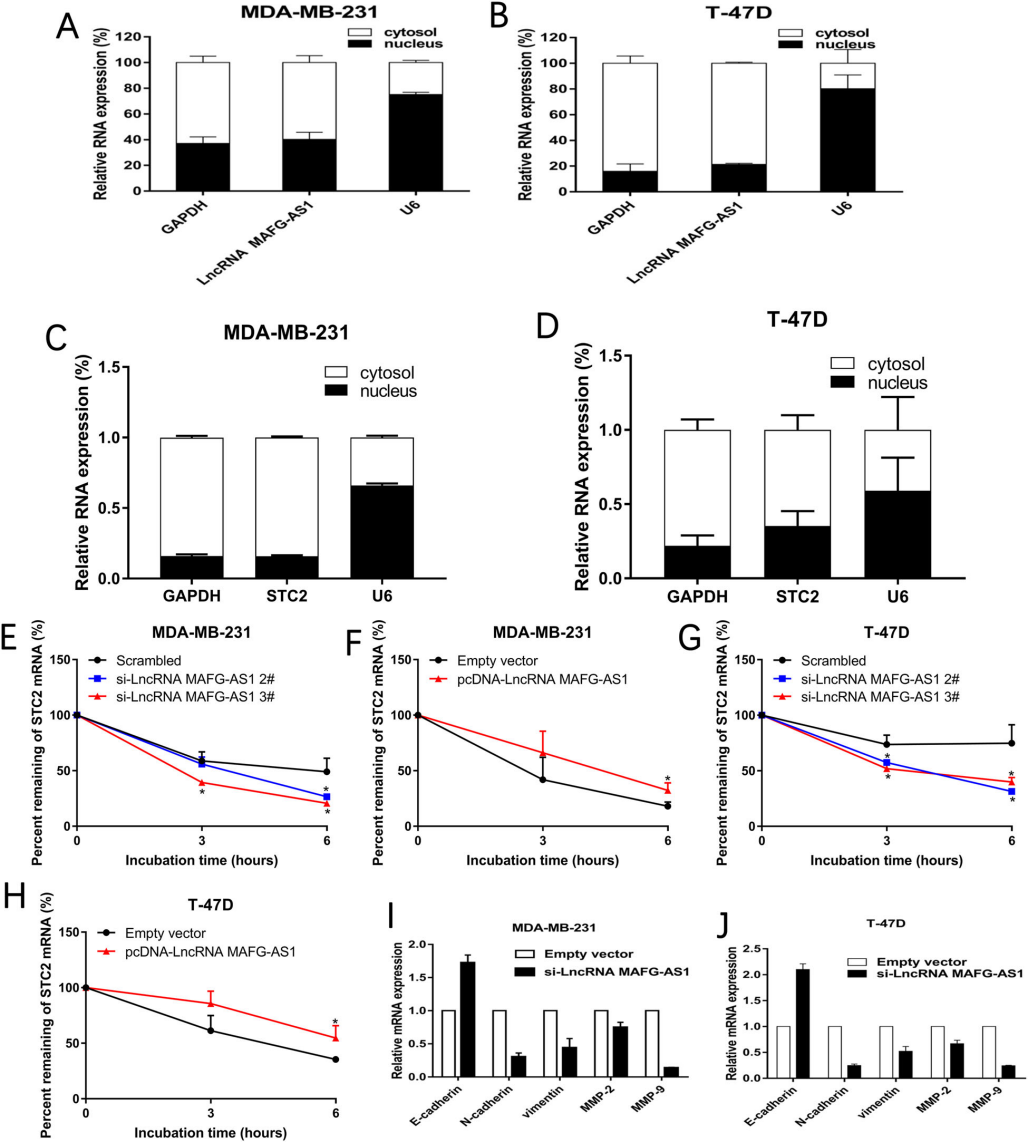
Prediction of potential mechanisms of oncogenic role of LncRNA MAFG-AS1. **A**, **B** LncRNA MAFG-AS1 expression levels in nuclear and cytoplasmic separation of MDA-MB-231 and T-47D cells were detected by qRT-PCR. GAPDH was used as cytoplasm control and U6 was used as nucleus control. **C**, **D)** STC2 mRNA expression levels in nuclear and cytoplasmic separation of MDA-MB-231 and T-47D cells were detected by qRT-PCR. GAPDH was used as cytoplasm control and U6 was used as nucleus control. **E**–**H** LncRNA MAFG-AS1 controled STC2 mRNA stability. RNA stability assays were performed in MDA-MB-231 and T-47D cells using Actinomycin D to disrupt RNA synthesis, and the degradation rates of the STC2 mRNA were measured over 6 h. **I**, **J)** Analysis of E-cadherin, N-cadherin, Vimentin, MMP-2, MMP-9 expression in MDA-MB-231 and T-47D cells treated with si-LncRNA MAFG-AS1. Values are shown as the mean ± s.d in three independent experiments. *P < 0.05, **P < 0.01.

To further understand the difference between cancer-promoting mechanisms of LncRNA MAFG-AS1 in nucleus and cytoplasm, and in order to study the functional mechanism of lncRNA MAFG-AS1, nuclear and cytoplasmic separation assay was exploited to detect its location in cells, which revealed that LncRNA MAFG-AS1 located in cytoplasm primarily in MDA-MB-231 and T-47D cells (Fig. 9A, B), and was colocalized with STC2 (Fig. 9C, D). Then we transfected si-LncRNA MAFG-AS1 into MDA-MB-231 and T-47D cells, then treated these cells with Actinomycin D (ACTD). Cells were collected every 3 h to extract RNA for qRT-PCR assay. We found that compared with the control group, the RNA degradation rate of STC2 was significantly increased after LncRNA MAFG-AS1 was knocked out, whereas overexpressed LncRNA MAFG-AS1 can slow down STC2 mRNA degradation (Fig. 9E–H). In conclusion, these results showed that LncRNA MAFG-AS1 regulated the expression of STC2 in breast cancer by promoting the stability of STC2 mRNA. A recent study found that lncRNAs played a crucial role in tumor cells invasion and metastasis as a regulator in epithelial-mesenchymal transition (EMT) and Matrix Metalloproteinases (MMPs). We detected the expression profile of EMT and MMPs related molecular markers and the results revealed that knockdown of LncRNA MAFG-AS1 induced the expression of E-cadherin and downregulated the expression of N-cadherin, Vimentin, MMP-2 and MMP-9 (Fig. 9I, J). To sum up, results from this study demonstrated that knockdown of LncRNA MAFG-AS1 suppressed breast cancer migration and invasion by modulating EMT and MMPs.

## Discussion

LncRNAs expressed abnormally in breast cancer cells, and participates in the important biological process including proliferation, apoptosis, invasion, and metastasis [18–21]. In spite of that the function and mechanisms of LncRNAs in breast cancer development and progression still remain unclear. Therefore, more studies to understand the relation of lncRNAs in the etiology of breast cancer are required, and more comprehensive and systematic studies for their regulatory network are under intense investigation, with clinical significance for early diagnosis, treatment, and improved prognosis in breast cancer.

In this study, bioinformatic tool was utilized to reveal that the expression level of LncRNA MAFG-AS1 in breast cancer tissues and found to be evidently elevated. Following qRT-PCR for detection demonstrated that expression of LncRNA MAFG-AS1 in breast cancer tissues was high, and that higher expression was connected to the higher malignant grade in clinic, which indicated the possibility that LncRNA MAFG-AS1 can be a potential molecular marker for diagnosis and prognosis. Results from in vivo and in vitro studies manifested that low-expressing LncRNA MAFG-AS1 could suppress the breast cancer cells’ proliferation, invasion, metastasis, and tumorigenic capacity and could induce its apoptosis. On the contrary, over-expressing LncRNA MAFG-AS1 promoted the breast cancer cells’ proliferation, invasion, and metastasis. All the results exhibited that LncRNA MAFG-AS1 played a critical role in the process of breast cancer as a carcinogen.

We screened for the STC2 as the targeted gene regulated by lncRNA MAFG-AS1 for the following study of mechanisms through transcriptome high-throughput sequencing. STC2 is a sort of human glycoprotein hormone, which was found in CS (corpuscles of Stannius) of bony fish by Stannius, made of cysteine, histidine and other amino acid residues [22]. STC2 is involved in the paracrine and autocrine regulation by expressing in tissues and participating in phosphate-regulating physiology, metabolism, regeneration, stress response, and development [23, 24]. Moreover, it was discovered that the expression of STC2 changed overtly in solid cancers such as colorectal cancer [25], nasopharyngeal carcinoma [26], endometrial carcinoma [27], gastric carcinoma [28], hepatocellular carcinoma [29], head and neck squamous cell carcinoma [30], which indicated that STC2 took effect in cancer development and progression, including promoting cancer cells invasion and metastasis, suppressing the cells apoptosis and so on. Our findings of the confirmatory test also demonstrated that STC2 may take part in the regulatory network of LncRNA MAFG-AS1. To summarize, it can be concluded that LncRNA MAFG-AS1 could promote breast cancer cells’ proliferation and metastasis by up-regulating targeted gene STC2. Mechanistically, it was mainly achieved by promoting the mRNA stability of STC2.

To further explore the specific mechanism how LncRNA MAFG-AS1 impacted breast cancer cells invasion and metastasis, we detected molecular expressions related to cells invasion and metastasis. It was found that EMT had a significant effect in process of cancer cells proliferation, invasion and metastasis, and the significant markers of EMT occurrence are the deficient expression of E-cadherin and elevated expression of N-cadherin and Vimentin [31, 32]. Hence, we further investigated the marker proteins relevant to cell invasion and metastasis in the breast cancer cells with knockdown of LncRNA MAFG-AS1, which showed that the suppression for breast cancer invasion and metastasis in the absence of LncRNA MAFG-AS1 may be associated with the EMT. Previous reports suggest that MMPs play a role in mediating cancer cell invasion and metastasis [33]. Our results from the qRT-PCR study revealed that knockdown of expression of LncRNA MAFG-AS1 in breast cancer cells could suppress the expression of MMP2 protein. Our hypothesis is supported by previous studies that knockdown of STC2 in AKT-ERK signaling pathway that could potentially suppress the process of EMT [34], and that up-regulating STC2 suppressed autophagy by downregulating the ratio of LC3 II / LC I and Beclin-1. Moreover, up-regulating STC2 could suppress cell apoptosis by activating AKT-ERK signaling pathway to suppress PARP, Bax and caspase 3 lysis proteins [33]. Results from our study indicated that knockdown of lncRNA MAFG-AS1 suppressed breast cancer cells migration, invasion and proliferation might by downregulating STC2 in AKT-ERK signaling pathway in breast cancer. However, the specific molecular mechanisms how LncRNA MAFG-AS1 regulates autophagy, EMT and cell apoptosis still need further investigation.

## Conclusions

The LncRNA MAFG-AS1 was found to be upregulated in breast cancer tissues and cells, and the upregulation of LncRNA MAFG-AS1 is related to some clinical parameters, which predicted the poor prognosis. Our study demonstrated that LncRNA MAFG-AS1 might promote breast cancer cell proliferation and metastasis by regulating the stability of STC2 mRNA. To summarize, lncRNA MAFG-AS1 could be a diagnostic markers and therapeutic target in the breast cancer, and it modulates the prognosis of breast cancer patients. However, the specific molecular mechanisms of LncRNA MAFG-AS1 in breast cancer development and progression still require additional evidence.

## Materials and methods

### Collection of breast cancer tissue and clinical data

Fifty-four pairs of breast cancer and adjacent normal breast tissues were obtained from the breast cancer patients undergoing surgery at the First Affiliated Hospital of Nanjing Medical University. These patients had not been treated locally or systematically before the surgery. All cases were confirmed by pathological diagnosis as breast cancer. Table 1 summarized the clinicopathological characteristics of all patients. The study had been approved by the relevant regulatory and independent ethics committees of the First Affiliated Hospital of Nanjing Medical University. Informed consent was obtained from each patient, all methods were carried out in accordance with relevant guidelines and regulations.

### Cell culture

Four human breast cancer cell lines (T-47D, MDA-MB-231, MDA-MB-468 and BT-474) and the normal human breast epithelial cell lines (MCF-10A) were purchased from the Chinese Academy of Sciences Biochemistry and Cell Biology Institute of Technology (Shanghai, China). T-47D and BT-474 cells were cultured in RPMI-1640 (GIBCO-BRL) medium, MDA-MB-231 and MDA-MB-468 cells were cultured in L-15 (GIBCO-BRL) medium. Both media were supplemented with 10% fetal bovine serum (FBS; ScienCell) and with 100 U/ml penicillin and 100 mg/ml Streptomycin (Invitrogen). In addition, the medium of T-47D should also be added 0.2 U/ml insulin. MCF10A cells were grown in Mammary Epithelial Cell Medium (MEpiCM; ScienCell). All cells were cultured at 37 °C with 5% CO_2_. Fresh medium was replaced every 2–3 days and cells were passaged when the cells confluence reached 80–90%.

### RNA extraction and qRT-PCR

Total RNA was extracted from BC tissues and cell lines using TRIZOL reagent (Invitrogen). cDNA was synthesized using the PrimeScrip-RT Reagent Kit (Takara). qRT-PCR was performed with the SYBR Green PCR kit (Takara) on an Applied Biosystems 7500 real-time PCR system. The results were normalized to GAPDH expression and analyzed using the 2^(−ΔΔCt)^ method. All of primers used in this study were summarized in Supplementary Table 1.

### Plasmid preparation

The full-length complementary DNA of LncRNA MAFG-AS1 was synthesized and sub-cloned into the pcDNA3.1(+) vector (Invitrogen) by Generay (Shanghai, China).T-47D and MDA-MB-231 cells were transfected with plasmid vector using Xtreme GENE HP DNA transfection reagent (Roche, Basel, Switzerland) according to the manufacturer’s instructions. Cells were harvested 48 h after transfection for qRT-PCR (Supplementary Table 1).

### RNA interference

Three individual LncRNA MAFG-AS1 siRNAs (si-LncRNA MAFG-AS1 1#, 2#, 3#), STC2 siRNA, and scrambled negative control siRNA (si-NC) were purchased from Invitrogen. T-47D and MDA-MB-231 cells were transfected with siRNAs using the Lipofectamine 2000 (Invitrogen, USA) according to the manufacturer’s instructions. The nucleotide sequences of siRNAs for LncRNA MAFG-AS1, STC2 were listed in Supplementary Table 2. Cells were harvested for qRT-PCR or western blot analysis 48 h after transfection.

### Cell-proliferation assays

Cell vitality was detected using the Cell Counting Kit-8 (CCK-8) assay kit (Bimake). Briefly, transfected cells were seeded into 96-well plates and incubated with CCK-8 reagent every 24 hours according to the manufacturer’s instructions. For colony formation assay, cells after transfection were cultured in 6-well plates, and after 14 days, the cells were fixed with methanol and stained with 0.1% crystal violet. Visible colonies were photographed. Cell proliferation was identified using Ethynyldeoxyuridine (EdU) assay following the manufacturer’s protocol of 5-ethynyl-2-deoxyuridine (EdU) labeling/detection kit (Ribobio, Guangzhou, China). The transfected cells were treated with 50 µM Edu labeling medium and incubated for 2 hours. Next, the cells were fixed with 4% paraformaldehyde and the cell membrane was permeated with 0.5% Triton X-100. Subsequently, cells were added with anti-Edu working solution and DAPI staining solution. Edu positive cells were identified and counted under fluorescent microscopy. The percentage of Edu positive cells was calculated from five random fields in three wells.

### Cell migration and invasion assays

For the migration assays, T-47D and MDA-MB-231 cells were transfected with si-LncRNA MAFG-AS1 or pcDNA-LncRNA MAFG-AS1, and were cultured in 24-well plates with an 8-mm pore size polycarbonate membrane (Corning Incorporated). For the invasion assays, cells in serum-free medium were placed into the upper chamber of an insert coated with Matrigel (Sigma-Aldrich) and medium containing 10% FBS was added to the lower chamber. After 24 h, the cells remaining on the upper chamber were wiped with cotton swabs, while cells on the lower membrane surface were fixed with methanol and stained with 0.1% crystal violet after 24 h incubation. Transwell migration assay was the same as invasion but with no Matrigel. Five fields of view were randomly selected in each well for counting. For the wound-healing assays, the cells were seeded in 6-well culture plates at 2 × 10^5^ cells per well. After the cells were adhered, pcDNA-LncRNA MAFG-AS1 (or empty vector) was transfected with the interference sequence or control sequence of MAFG-AS1. Two to four straight lines were drawn in the six-well plate using a 10 µl pipette tip, the floating cells were washed with PBS. Cell gaps at the starting point were captured under a microscope. The six-well plate was then placed back inside the incubator for 24-48, and the progress of the cell gap closure was closely monitored. Images were captured, and the intercellular space was measured using a software. The treatment group was compared with the control group while analyzing the data.

### Tumor Induction assay in a nude mouse model

Female BALB/c-nude mice (4-weeks-old) which were purchased from Charles River (Zhejiang, China) were maintained under specific pathogen-free (SPF) conditions and manipulated according to the protocols approved by the Shanghai Medical Experimental Animal Care Commission. T-47D cells were stably transfected with si-LncRNA MAFG-AS1 and empty vector and harvested 24–48 h later. Cells were then washed with phosphate-buffered saline (PBS), and re-suspended at a concentration of 1 × 10^8^ cells/ml. A total of 100 μl of suspended cells was bilaterally subcutaneously injected into a single side of the armpit of each mouse. A total of 10 nude mice and the left side was the si-NC group and the right side is the si-LncRNA MAFG-AS1 group. Tumor growth was continuously monitored at an interval of every 5 days, and tumor volumes were calculated using the equation *V* = 0.5 × *D* × *d*^2^ (*V* represents volume, *D* represents longitudinal diameter, *d* represents latitudinal diameter). At 28 days post-injection, mice were euthanized and the subcutaneous growth of each tumor was examined. This study was performed strictly in accordance with the recommendations in the Guide for the Care and Use of Laboratory Animals of the National Institutes of Health. The protocol was approved by the Animal Ethical and Welfare Committee of Nanjing Medical University (approval number:1601248-4). The primary tumors were immune-stained for Ki-67 (MX006, MXB, Fuzhou, China) as previously described. Each experiment was repeated three times.

### Flow-cytometry (FCM) analysis

Flow Cytometric analysis T-47D and MDA-MB-231 cells were transfected with si-LncRNA MAFG-AS1 or scrambled and were harvested 48 h after transfection by trypsinization. Cells were stained with FITC Annexin V, and propidium iodide (PI) by using the FITC Annexin V Apoptosis Detection Kit (BD Biosciences) following the manufacturer’s protocol. The cells were analyzed by flow cytometry (FACScan®; BDBiosciences) with CellQuest software (BD Biosciences). The cells were classified into viable, dead, early apoptotic, and apoptotic cells, and then the ratio of early apoptotic cells was compared with the control for each experiment.

### Nuclear and cytoplasmic separation assay

The separation of nuclear and cytosolic fractions was performed using the PARIS Kit (Life Technologies) according to the manufacturer’s protocol.

### Transcriptome high-throughput sequencing

Plant T-47D cells in a six-well plate and lyse with 1 ml Trizol per well 24 h after transfection with si-LncRNA MAFG-AS1. Retain 200ul of lysate to extract total RNA to detect interference efficiency and the other 800ul lysate was sent for high-throughput sequencing.

### Statistical analysis

The Student’s *t* test (two-tailed), one-way ANOVA and Chi-square test were conducted to analyze in vitro and in vivo data by SPSS 17.0 software. *P* values less than 0.05 were considered as significant.

## Supplementary information

Supplementary Figure 1, Supplementary Table 1, Supplementary Table 2
