# Supplementary material for: Long non-coding RNA MAFG-AS1 promotes proliferation and metastasis of breast cancer by modulating STC2 pathway
Source: Cell Death Discov. 2022 May 5;8:249. doi: 10.1038/s41420-022-01043-z (PMC9072673; doi:10.1038/s41420-022-01043-z)
Supplement: Supplementary file 1 — Supplementary Figure 1, Supplementary Table 1, Supplementary Table 2 [file 41420_2022_1043_MOESM1_ESM.docx]

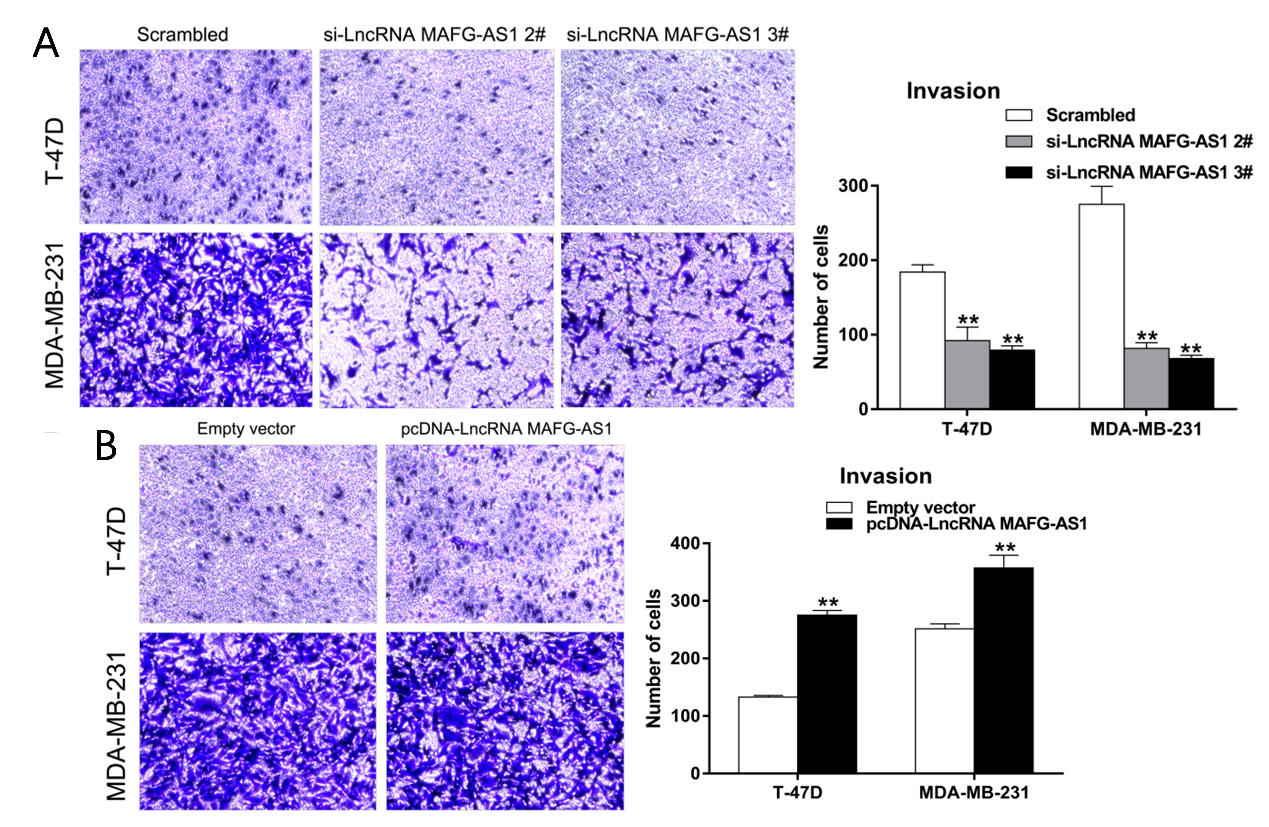


**Supplentary figure 1** (A and B) Transwell invasion assays with matrigel were used to investigate the changes in migratory abilities of BC cells after transfection with si- LncRNA MAFG-AS1 or pcDNA-LncRNA MAFG-AS1. *P < 0.05 and **P < 0.01.

**Supplentary table 1: Primers for qRT-PCR.**

| Name of gene | Pre-primers (sequence 5’ to 3’) | Post-primers (sequence 5’ to 3’) |
| --- | --- | --- |
| MAFG-AS1  SLC25A6  STC2  TUBA1B  SERPINA6  CBX5  STK17A  CDK1  GAPDH | CGGGAGGAAGATAAACGGGG  CCACTCAAGCCCTCAACTTC  CCGTGAGGTCTCTGTCGAA  GACCTTGTGTTGGACCGAAT  GGCCTGGCTATACTGGACAA  ACTTGGATTGCCCTGAGCTA  TCTGAGTCGGCTGTTGATTTC  AGGTCAAGTGGTAGCCATGAA  GGGAGCCAAAAGGGTCAT | TGACCACGGGAACACCTTCAG  CGCTCTGTGCCTGACTTTC  TGAGTTGACGGATAAGGATGC  GGTGCTGGGTAAATGGAGAA  TTTGGGACTCAAGGCCACTA  TCACCACAGGAATCTGTTGC  GGGGTGCTTTAGACATTCTTCA  TGTACTGACCAGGAGGGATAGAA  GAGTCCTTCCACGATACCAA |

**Supplentary table 2: si RNA sequence.**

| Name | Sequence (5’ to 3’) |
| --- | --- |
| si-LncRNA MAFG-AS1 1# | AUCUCCAACAAGCAGCGAAGUCUCC |
|  | GGAGACUUCGCUGCUUGUUGGAGAU |
| si-LncRNA MAFG-AS1 2# | ACCAGAACCGCGAAAGGCUACUGUA |
|  | UACAGUAGCCUUUCGCGGUUCUGGU |
| si-LncRNA MAFG-AS1 3# | GGAGUCAGGGCAAUUCCAACCAAGA |
|  | UCUUGGUUGGAAUUGCCCUGACUCC |
| si-STC2 | GACUGCCUGGUGAAGAUCACCAAGU |
|  | ACUUGGUGAUCUUCACCAGGCAGUC |
| Scrambled | UUCUCCGAACGUGUCACGUTT |
|  | ACGUGACACGUUCGGAGAATT |
